# Transgenic Bt Cotton Does Not Disrupt the Top-Down Forces Regulating the Cotton Aphid in Central China

**DOI:** 10.1371/journal.pone.0166771

**Published:** 2016-11-21

**Authors:** Yong-Sheng Yao, Peng Han, Chang-Ying Niu, Yong-Cheng Dong, Xi-Wu Gao, Jin-Jie Cui, Nicolas Desneux

**Affiliations:** 1 Department of Entomology, China Agricultural University, Beijing, China; 2 College of Plant Science, Tarim University, Alar, China; 3 Hubei Insect Resources Utilization and Sustainable Pest Management Key Laboratory, College of Plant Science and Technology, Huazhong Agricultural University, Wuhan, China; 4 INRA (French National Institute for Agricultural Research), Université Nice Sophia Antipolis, CNRS, UMR 1355–7254 Institut Sophia Agrobiotech, Sophia Antipolis, France; 5 State Key Laboratory of Cotton Biology, Institute of Cotton Research CAAS, Anyang, China; CSIRO, AUSTRALIA

## Abstract

Top-down force is referred to arthropod pest management delivered by the organisms from higher trophic levels. In the context of prevalent adoption of transgenic Bt crops that produce insecticidal Cry proteins derived from *Bacillus thuringiensis* (Bt), it still remains elusive whether the top-down forces are affected by the insect-resistant traits that introduced into the Bt crops. We explored how Bt cotton affect the strength of top-down forces via arthropod natural enemies in regulating a non-target pest species, the cotton aphid *Aphis gossypii* Glover, using a comparative approach (i.e. Bt cotton *vs*. conventional cotton) under field conditions. To determine top-down forces, we manipulated predation/parasitism exposure of the aphid to their natural enemies using exclusion cages. We found that the aphid population growth was strongly suppressed by the dominant natural enemies including Coccinellids, spiders and Aphidiines parasitoids. Coccinellids, spiders and the assemblage of other arthropod natural enemies (mainly lacewings and Hemipteran bugs) are similarly abundant in both plots, but with the parasitoid mummies less abundant in Bt cotton plots compared to the conventional cotton plots. However, the lower abundance of parasitoids in Bt cotton plots alone did not translate into differential top-down control on *A*. *gossypii* populations compared to conventional ones. Overall, the top-down forces were equally strong in both plots. We conclude that transgenic Bt cotton does not disrupt the top-down forces regulating the cotton aphid in central China.

## Introduction

In terrestrial ecosystems, bottom-up and top-down forces in structuring arthropod communities have been extensively documented. Top-down force is characterized as the population suppression of herbivorous arthropods caused by predation, parasitism or infection exerted by the organisms from higher tropic levels i.e. natural enemies [1–5]. However, the strength of top-down forces via these biological interactions are susceptible to various biotic and/or abiotic factors [6–8], and they can even interact with bottom-up forces [9].

Top-down force is referred to arthropod pest management in the context of biological control in agro-ecosystems [10]. Within “plant-herbivores insect-natural enemy arthropod” tri-trophic interactions, however, top-down forces are influenced by various biotic factors such as plant features in terms of food/shelter and resistant traits [11,12], or abiotic factors such as fertilization regimes [13–15]. In order to enhance top-down forces, various habitat manipulation strategies have been proposed to attract natural enemies and/or promote their performance in suppressing the pest populations [12,16]. Specifically, these practices have been advised in the “Push-Pull strategy” [17]. In such strategies, the options have largely relied on enhancing the biocontrol services of natural enemies through manipulation of physical and chemical features of habitat (i.e. external factors). However, it is acknowledged that the manipulation and/or selection of innate traits of plants [18,19], as well as insect-resistance traits that are introduced into the plants, may also impact the arthropod natural enemy community and thus the efficacy of top-down forces.

In modern agriculture, insect-resistant transgenic crops—mostly produce *Bacillus thuringiensis* (Bt) proteins—have been increasingly adopted worldwide [20]. Bt crops have been and continue to be effective tools offering considerable management of several major pest guilds, and they have been regarded as one of the most cost-efficient and environmentally sound strategies [21,22]. While Bt crops are developed to manage target phytophagous pests, the unintentional effects of Bt crops on non-target phytophagous insect and arthropod natural enemies [23–27] and specifically the impacts on their behaviors [28] should not be underestimated. These issues have raised the concern on the ecological compatibility between the crop insect-resistance traits and biological control delivered by those biocontrol agents [29].

The Yangtze River Valley Cotton-planting Zone (YRZ) is one of the largest cotton-planting regions in China. In this region, Bt cotton has long been commercially used. The cotton aphid, *Aphis gossypii* Glover (Hemiptera: Aphididae) is considered as a secondary pest in YRZ as it is not targeted by *Bt* toxins as other aphid species [30]. Despite that cotton aphids have shown continuous decline in their seasonal population density in Bt cotton fields in the past two decades in China [6], cotton aphid outbreaks occasionally occur and reach economically damaging levels in some areas [31], which was likely due to particular weather conditions (e.g. less rainfall during the aphid population-growth season) or pesticide resistance [32]. We have documented strong top-down forces in regulating *A*. *gossypii* in Bt cotton field [10]; such top-down forces have been primarily offered by Coccinellids and Aphidiine parasitoids. It still remains elusive whether the top-down forces have been influenced by the insect-resistant traits introduced into Bt cotton cultivars. This issue becomes extremely relevant since the unintentional direct and indirect effects of Bt crops on beneficial arthropods (e.g. natural enemies) have been recently highlighted [27,28]. In addition, while direct impact of Bt cotton on the cotton aphid is unlikely [31], other co-occurring arthropods may indirectly affect *A*. *gossypii* via plant-mediated indirect interactions. A recent study has documented that such interaction can be largely mediated by the presence of Bt proteins [33]. That study proposed that Bt cotton may benefit Bt-insensitive herbivores (e.g. aphids), which might link to the decreased inducible chemical defenses in Bt cotton due to lack of Lepidopteran pests feeding.

As a departure of our previous study [10], we further explored how Bt cotton affect the strength of top-down forces in regulating *A*. *gossypii* populations in the field. We designed a comparative study using a Bt cotton cultivar combining two insect-resistance genes *Cry1Ac* and *CpTI* (Cowpea Trypsin Inhibitor), and its near-isogenic cultivar as control. The objective is to enhance the knowledge on ecological compatibility between transgenic insecticidal traits in cotton and biocontrol services offered by arthropod natural enemies in cotton agro-ecosystems.

## Material and Methods

### Cotton varieties

Seeds of Bt cotton (“CCRI 41”, hereafter named “Bt cotton”), producing Cry1Ac and CpTI (Cowpea Trypsin Inhibitor), and those of the corresponding non-Bt near-isoline (“CCRI 23”, hereafter named “conventional cotton”) were obtained from College of Plant Sci & Tech, Huazhong Agricultural University. The CpTI expressed in tobacco plants enhances the insect resistance to a variety of insect pests as it has anti-metabolic activity to the insects from the orders Lepidoptera, Coleoptera, and Orthoptera [34]. We assume that *A*. *gossypii* is not targeted by the two types of insect resistance in the cultivar CCRI 41. These two cultivars have been extensively used in our previous studies investigating the potential side effects of Bt cotton on non-target arthropods [35–37].

### Aphid colony

Naturally occurring cotton aphids were collected in May 2013 from a cotton field at the Experimental Station of Huazhong Agricultural University (HZAU), Wuhan, China. The aphid colony was reared on the non-Bt cotton seedlings, i.e. cultivar CCRI 23, under laboratory conditions (28 ± 1°C, RH 70 ± 10%, 14 h light). The aphid colony lived healthy on those seedlings and no yellow dwarf was observed. The colony was subsequently used as the source for artificial infestations of cotton plants in the field experiments.

### Field experiment setup

The field experiment was conducted at the Experimental Station of HZAU. The field was previously cultivated free of insecticide application and only the routine agronomic practices (e.g. tillage) were applied. Bt cotton and conventional cotton seedlings were planted in a 1.43-ha field on 12^th^ May. Regarding the seedling density, the sowing distance was 40 cm in each row with a distance of 30 cm between the two rows within each ridge. The distance among the ridges was 80 cm. The foliar fertilization of urea (two times of spray at concentrations of 0.5% and 1% on 20^th^ May and 14^th^ June, respectively) and monopotassium phosphate (active gradient with 750 g/ ha) was uniformly applied to both cultivars (Hubei Yihua Chemicals Ltd., China). Other routine practices were performed except for pesticides application [10]. Weed control relied on hand hoeing at four different dates throughout the experiment. Irrigation was conducted once during the dry period in June and ditches were made to facilitate drainage during the rainy season in July.

The cage restriction technique was designed following the methods described in our previous study [10]. All the cages were made of bamboo sticks (2m x 2m x 2m, length x width x height) covered by nylon mesh netting with openings of 530×530μm. Three different treatments were applied using restriction cages: (i) “exclusion cages”, four upright bamboo sticks completely coved by mesh in which aphids were fully-protected from arthropod natural enemies (predators and parasitoids); (ii) “sham cages”, built with the netting mesh but included a 40-cm high opening in the middle and the bottom of the cage respectively. This treatment was set up to assess the possible disruptive effect of cages (e.g. mesh and bamboo sticks) on the activity of natural enemies and aphid population dynamics; (iii) “no cage”, the completely open area (named “open field” thereafter) which consisted of four bamboo sticks standing upright and a tape surrounding them as a cue for sampling range. To avoid the effects of ground-dwelling predators, a plastic barrier (around 20 cm buried in the soil) was connected to the nettings of each exclusion and restriction cage after the study [38].

During 18^th^ June and 20^th^ June, we set up two blocks with one for each cultivar, i.e. either conventional or Bt cotton. The two blocks were in the same field separated by a distance of 50 meters. Each block included four plots. Within each of these plots three sub-plots were designed, with one for each of the three treatments (exclusion cage, sham cage and no cage) randomly allocated. We kept a distance of 10 meters among the four plots and 4 meters among the sub-plots. Four plants were covered within each cage type which allowed the full growth of cotton within the cage [10]. One side of the exclusion cage was sewed with a zipper allowing entry for sampling.

### Arthropod population dynamics

Prior to artificial infestation of aphids, any resident aphids and other insects were removed by hands and aspirators within the plots that covered by cages. On 21^th^ and 22^nd^ June when cotton plants were in flower bud stage with an average plant height of 45 cm, each plant within the plot was artificially infested by ten aphid adults choosing one leaf from the top of the plant. The aphids were susceptible to wounding during the infestation. Thus the number of aphids was checked in the following two days and new aphids were refreshed when necessary. Starting from 28^th^ June until 25^th^ August, the aphids and/or their natural enemies within each treatment were recorded. The whole body of the four plants in each plot was visually checked to count the arthropods. The sampling procedure did not cause any physical damage to the plants. Flying species were firstly recorded by gently shaking the plants. We counted the adults and larvae of ladybeetles, adults of spiders, and Aphidiine parasitoid mummies, as well as other arthropod natural enemies mainly including lacewings, Hemipteran bugs, hoverflies, and mantis. All the recorded species were identified to family or species level. The identification followed the experience obtained from our previous study [10], as well as the instructions from a guidebook specializing on cotton pest and disease identification and management in China [39]. For identification of aphid parasitoids, the un-merged parasitoid mummies, i.e. pupae stage of the parasitoid, were counted (with black- and tan-colored mummies belonging to the Aphelinidae and Aphidiinae parasitoid families, respectively). Mummy samples were collected for further identification of parasitoids following the identification keys previously reported [40–45]. The sampling was conducted every 5–7 days from noon to 6 pm for each sampling date [10].

### Data analyses

We tested the effect of “cultivar” (Bt cotton *vs*. conventional cotton), “cage type” (exclusion cage *vs*. sham cage *vs*. open field), “date” (12 sampling dates) as well as their two-by-two interactions (if applicable) on the density of cotton aphid and four arthropod natural enemy groups: Coccinellids, spiders, parasitoid mummies, and other natural enemies. Since the assemblage of Coccinellid adults and larvae acted as generalist predators, the counts of the adults and larvae were pooled for the analysis. The data were fitted in a GEE GLM (Generalized Estimating Equations Generalized Linear Model) based on a Poisson error and a log-link function with repeated measurements (“sampling date” as repeated factor). For the datasets of natural enemies, we failed to run the model by considering all the two-by-two interactions due to the numerous zero count values. We then analyzed the data using a more parsimonious model to remove some of the main effect or interactions that failed to give signs in the model. Such a parsimonious model has often been used to run GEE GLM on the dataset where numerous zero data exist. The main effect of “date” and its interaction with other factors on parasitoid mummies were not able to be tested due to the numerous zero counts. All the data were processed using R software (R Version 3.2.2, R Core team, Vienna, Austria, 2009) [46].

## Results

A summary of total counts of dominate arthropods in experimental plots of Bt cotton and conventional cotton were provided (Table 1). The natural enemy guild was dominated by Coccinellids, spiders as well as Aphiidiines parasitoids (tan-colored mummies). The other natural enemies mainly consisted of lacewings, Hemipteran bugs and mantis.

**Table 1:** A summary of sampled arthropods in the field survey. Total counts of dominant arthropods in the experimental plots of Bt cotton or conventional cotton at HZAU experimental station (Wuhan, China) from late June to late August in 2013.

Guild	Group/Species	Bt cotton	Conventional cotton
The pest	Cotton aphids	28022	31454
Key arthropod natural enemies	Ladybeetles^a^	171	193
	Spiders^b^	114	118
	Aphidiine parasitoids^c^	155	221
Other arthropod natural enemies	Lacewings^d^	81	79
	Hemipteran bugs^e^	70	88
	Mantis^f^	6	11

^a^ includes *Propylaea japonica* Thunberg (67.8%), *Harmonia axyridis* Pallas (17.0%), *Coelophora saucia* Mulsant (11.2%), and other unidentified species.

^b^ includes *Ebrechtella tricuspidata* Fabricius (48.2%), *Hylyphantes graminicola* Sundevall (36.7%), and other unidentified species.

^c^ includes Lysiphlebia japonica Ashmead (87.3%), *Biondoxys indicus* Subba Rao & Sharma, *Ischnojoppa luteator* Fabricius and other unidentified species.

^d^ includes *Chrysopa pallens* Rambur (84.6%) and *Chrysoperla sinica* Tjeder (15.4%).

^e^ includes *Orius similis* Zheng (43.0%) and other species from Miridae and Nabidae families.

^f^ refers to *Hierodula saussurei* Kirby

**Fig 1:**
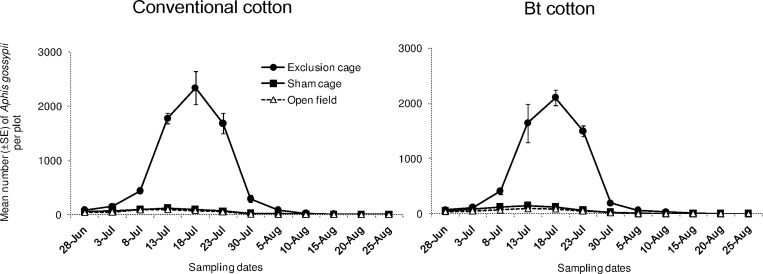
Population dynamics of cotton aphids. Mean numbers (±SE) of *A*. *gossypii* per plot in exclusion cages, sham cages and open field plots at HZAU experimental station (Wuhan, China) from late June to late August in 2013.

*Aphis gossypii* densities differed significantly among sampling dates (Fig 1; “date”: χ^2^ = 3446, df = 11, *P* < 0.001) and among cage types (“cage type”: χ^2^ = 255.1, df = 2, *P* < 0.001). However, the overall population density of *A*. *gossypii* did not differ between the two cultivars (“cultivar”: χ^2^ = 1.600, df = 1, *P* = 0.204). Furthermore, the effects of different cages on their density did not differ between the two cultivars (“cage type x cultivar”: χ^2^ = 0.100, df = 2, *P* = 0.961). In both cultivars, the population density peak of *A*. *gossypii* reached over 100-fold growth in exclusion cages compared to the sham cages and open field plots.

**Fig 2:**
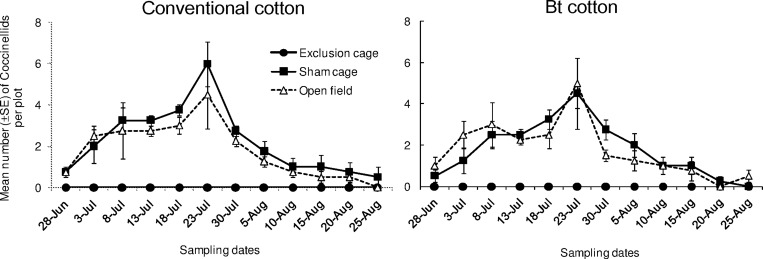
Population dynamics of ladybeetles. Mean numbers (±SE) of Coccinellids per plot in exclusion cages, sham cages and open field plots at HZAU experimental station (Wuhan, China) from late June to late August in 2013.

Coccinellids densities differed significantly among the sampling dates (Fig 2; “date”: χ^2^ = 100.1, df = 11, *P* < 0.001) and among cage types (“cage type”: χ^2^ = 24.68, df = 2, *P* < 0.001). However, the overall Coccinellids population density did not differ between the two cultivars (“cultivar”: χ^2^ = 2.616, df = 1, *P* = 0.106). The effect of cage type on Coccinellids densities were similar on both cultivars (“cage type x cultivar”: χ^2^ = 0.043, df = 2, *P* = 0.979). In sham cages and open field plots, the counts of this group followed a similar trend in both cultivars as a whole. The density of Coccinellids increased gradually and peaked at 6^th^ sampling date and declined until the end of the survey.

**Fig 3:**
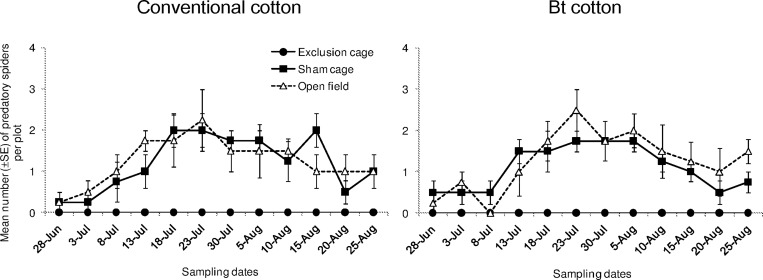
Population dynamics of spiders. Mean numbers (±SE) of spiders per plot in exclusion cages, sham cages and open field plots at HZAU experimental station (Wuhan, China) from late June to late August in 2013.

Densities of spiders differed significantly as function of the sampling dates (Fig 3; “date”: χ^2^ = 67.60, df = 11, *P* < 0.001) and among cage types (“cage type”: χ^2^ = 16.33, df = 2, *P* < 0.001). However, their overall population density did not differ between the two cultivars (“cultivar”: χ^2^ = 1.646, df = 1, *P* = 0.200). The effect of cage type on spider densities was similar on both cotton cultivars (“cultivar x cage type”: χ^2^ = -86.46, df = 2, *P* = 1.000). In sham cages and open field plots, we observed relatively higher densities of Coccinellids at 5^th^ and 6^th^ sampling date. Unlike Coccinellids populations, they did not show any evident density peak and they were more evenly distributed on a temporal scale.

**Fig 4:**
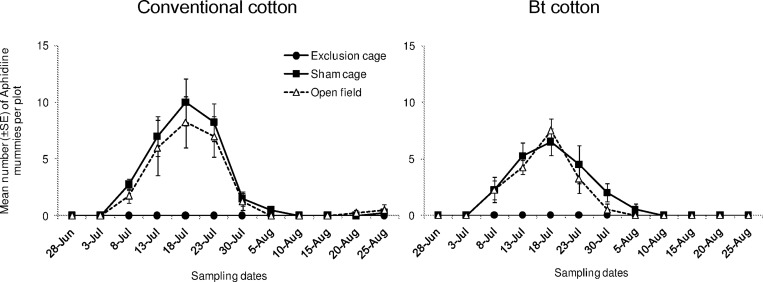
Population dynamics of aphid parasitoids. Mean numbers (±SE) of Aphidiine mummies per plot in exclusion cages, sham cages and open field plots at HZAU experimental station (Wuhan, China) from late June to late August in 2013.

Aphidiines mummies (tan-colored) were most commonly observed whereas Aphelinidae mummies (black-colored) were barely found during the whole study (with six in total). Their densities varied significantly among cage types (Fig 4; “cage type”: χ^2^ = 14.25, df = 2, *P* < 0.001) and showed a similar trend in both Bt and conventional cultivars (“cage type x cultivar”: χ^2^ = 0.117, df = 2, *P* = 0.943). However, Aphidiine mummies were much less abundant in Bt cotton compared to conventional cotton plots (“cultivar”: χ^2^ = 7.310, df = 2, *P* = 0.007). Specifically at 5^th^ and 6^th^ sampling date, Bt cotton plots harbored much less (an average of 4 and 6 mummies respectively) than conventional cotton plots (7 and 9 mummies respectively). The overall densities of Aphidiine mummies fluctuated among a given period of sampling dates (from 3^rd^ and 7^th^ sampling date) whereas they were rarely found in other sampling dates.

**Fig 5:**
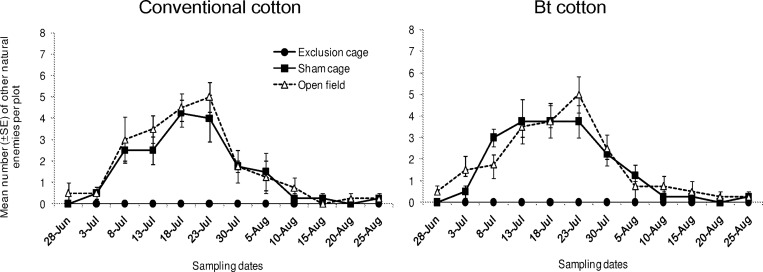
Population dynamics of other natural enemies. Mean numbers (±SE) of other natural enemies per plot in exclusion cages, sham cages and open field plots at HZAU experimental station (Wuhan, China) from late June to late August in 2013.

The assemblage of other natural enemies sampled mainly included lacewings, omnivorous Hemipteran predators, e.g. the bugs belonging to the Miridae, Nabidae and Anthocoridae families, as well as mantis (Table 1; S1 Fig). The sum of densities of these natural enemies differed significantly among sampling dates (Fig 5; “date”: χ^2^ = 146.5, df = 11, *P* < 0.001) and among cage types (“cage type”: χ^2^ = 5024, df = 2, *P* < 0.001). However, their overall population density did not differ between the two cultivars (“cultivar”: χ^2^ = 0, df = 1, *P* = 1.000). The effect of cage type on the densities of these natural enemy arthropods was similar on both cotton cultivars (“cultivar x cage type”: χ^2^ = 1.566, df = 2, *P* = 0.457). In sham cages and open field plots, we observed relatively higher densities of those arthropods from 3^rd^ and 7^th^ sampling date. They were found in relatively low abundance during the last four weeks.

## Discussion

Our study provided empirical evidence that transgenic Bt cotton did not disrupt the top-down forces regulating the cotton aphid *A*. *gossypii* in central China. The aphid population growth was strongly suppressed by the presence of natural enemy guild (mainly Coccinellids, spiders and parasitoids). Furthermore, such top-down management was equally strong in both Bt and conventional cotton cultivars. Except that parasitoid mummies are less abundant in Bt cotton plots compared to conventional cotton plots, Coccinellids, spiders and the assemblage of other arthropod natural enemies are similarly abundant in both plots.

We demonstrated strong top-down force exerted by natural enemies on cotton aphid populations. With abundant natural enemies in the open field plots, the aphid population growth was quite limited when compared to aphid populations in exclusion cages i.e. without attack by natural enemies. In addition, aphid populations were kept at relatively low levels in the open field plots throughout the season (despite small population increase from 8th July to 23rd July, Fig 1). However, the aphid population densities did not differ between Bt and conventional cotton plots in open fields. This result does not support the hypothesis that Bt crops may benefit the population growth of sap-feeding herbivores due to decreased damage by sensitive lepidopteran pests [33]. We assume such an inconsistence may be caused by other unknown ecological factors in the agro-ecosystems that deserve further investigations.

Coccinellids have been found to respond numerically to the seasonal population dynamic of *A*. *gossypii*. These results corroborate our previous study which has recognized them as predominant biocontrol agents for management of the aphid in Bt cotton field [9]. Similar top-down forces via Coccinellids have been observed on other aphid species in other cropping systems [38,47,48]. In our study, *Propylaea japonica* Thunberg was the most abundant species within Coccinellids group, with *Harmonia axyridis* Pallas and *Coccinella septempunctata* Linnaeus less abundant. *Propylaea japonica* is a well-known generalist predator of *A*. *gossypii* [10,31,49,50]. This species can colonize the cotton field during the early cotton seeding stage when aphid populations start to grow. Besides the aphid they favor, these generalist predators include other prey items (i.e. spider mites and whiteflies) and non-prey food into their diets, such as honeydew and nectar (observation by YSY). Despite its predominance, i.e. 67.8%, *P*. *japonica* population has not been independently analyzed in our study. This is mainly for two reasons. Firstly, all these Coccinellidae species represent a functional guild which can effectively suppress the establishment and subsequent population growth of aphids in the whole season. Secondly, the population dynamics of *P*. *japonica* alone was quite similar to the one when all the three species were pooled together. Spiders, another important group of generalist predators, were also found abundant during the season. However, unlike Coccinellids and parasitoids, they did not respond numerically to the aphid population. One explanation could be that *A*. *gossypii* only consists of a small part of diet for the spiders. Still, they can contribute to slowing down aphid population growth when the aphids are at low density. A molecular approach may be useful to quantify the actual predation rate occur in the field [51].

High densities of Aphidiidae parasitoids mummies, mainly *Lysiphlebia japonica* Ashmead and *Binodoxys indicus* Subba Rao & Sharma, were found most abundant. Density of the mummies showed a numerical response to the aphid density, which was consistent to our previous report [10]. However, our current study does not examine the single role of the parasitoids in management of *A*. *gossypii* which has been identified using restriction cages [10]. Combined impacts of both the generalist predators and specialist parasitoids on *A*. *gossypii* are likely to occur, even though the intraguild predation pressure on the parasitoids mummies was likely to occur in the open field [52].

When comparing the population dynamics of natural enemies between Bt and conventional cotton plots, however, we found a lower abundance of parasitoid mummies in Bt cotton plots compared to conventional ones. At least one hypothesis can be evoked to explain such results. The lower abundance of the parasitoid species may be due to the differences in herbivore-induced volatiles between Bt cotton and conventional cotton. Compared to Bt cotton, conventional cotton that heavily damaged by target pests may release quantitatively and qualitatively different plant volatiles and thus may make them more attractive to the parasitoid wasps. Indeed, lepidopteran pests (e.g. *Agrotis ypsilonn* Rottemberg and *Spodoptera litura* Fabricius from the Noctuidae family) were abundant (10–20 larvae per plant during the middle of the season, observation by YSY) in conventional cotton plots whereas they were scarcely observed in Bt cotton plots. Nevertheless, previous studies did not detect any measurable difference in parasitoid foraging between slightly-infested Bt and heavily-infested non-Bt plants from the perspective of changes in profile of organic volatile compounds (VOCs) [53–56]. Besides the effects of plant-emitted volatiles, other possible mechanisms underlying the low density of parasitoids in Bt cotton field deserves further research. Without replication of conventional and Bt cotton crops (fields) in our study, it is possible that differences in parasitoid numbers resulted from other factors, such as local landscape features. The meta-analysis by Chaplin-Kramer et al.[57] has shown that landscape features on a narrower spatial scale is more likely to influence specialist natural enemies (e.g. aphid parasitoids), whereas generalist predators responded more strongly to landscape features at broader scales. In such a small-scale landscape in our study, we assume that the difference in parasitoid abundance between the conventional and Bt cotton crops might behave slightly differently in other fields showing different landscape features.

The generalist predators and other arthropod natural enemies showed similar abundance as well as similar patterns of population dynamics between Bt and conventional cotton plots. Population densities of the assemblage “other natural enemies” mainly including Syrphidae, Chrysopa (lacewings), Hemiptera (Miridae, Nabidae and Anthocoridae) did not differ between the two cotton cultivars. Some species from this group are omnivorous predators. Even though they frequently feed on plants by inserting their stylets into plant tissues and sucking liquid content and liquefied materials through enzymatic degradation in salivary [58], Bt toxins are not detectable in their bodies [59]. We assume that the impact of Cry protein in Bt cotton on these predators was negligible. In addition, our results support the hypothesis that response of generalist natural enemies to natural habitat tends to occur at large spatial scales [57], not at small scale in the present study.

Within-year seasonal declines of aphid abundance are often correlated with a high level of predation and parasitism [60,61]. Such strong top-down forces have been shown in our study. However, it appears that the change in parasitoid abundance alone was not strong enough to result in different regulation strength on *A*. *gossypii* populations between Bt and conventional cotton plots. In agro-ecosystems, aphids have complex population dynamics often characterized by wide variations of population density, both on a temporal and spatial scale [62]. Their spatial-temporal distribution patterns can be influenced by many other organisms, not limited to natural enemies which contribute to the suppression of aphid populations [6]. For example, it remains unclear how *A*. *gossypii* population could be affected by the changes in plant suitability (e.g. nutritional quality and defensive traits) induced by the assemblage of co-occurring phytophagous pests (e.g. *Bemisia tabaci* Gennadius, *Empoasca biguttula* Shiraki and *Tetranychus cinnabarinus* Boisduval). Indeed, it has been recently documented that changes in secondary metabolisms, e.g. reductions in gossypol and tannin contents in Bt cotton, have benefited spider mites showing decreased generation time and increased fertility [63].

In conclusion, our data demonstrated that transgenic Bt cotton did not disrupt the top-down forces contributing to the regulation of cotton aphid *A*. *gossypii* in central China. Our current experimental design may not be optimal to compare Bt cotton and conventional cotton due to the limited availability of fields. The experimental design should include several field plots in future comparative studies. One prospective subject could be examining whether the foraging behavior of parasitoid wasps are disrupted in Bt cotton plots in terms of plant-emitted VOCs compared to conventional cotton plots [55,56]. It is also interesting to study how the genetic engineering affect the indirect interactions between cotton aphid and other herbivores, which can be mediated by plant and/or generalist predators [64,65].

## Supporting Information

S1 Fig Population dynamics of lacewings and Hemipteran bugs. Mean numbers (±SE) of lacewings and Hemipteran bugs per plot in exclusion cages, sham cages and open field plots at HZAU experimental station (Wuhan, China) from late June to late August in 2013.
(PDF)
