# Supplementary figures and images for: Transgenic Bt Cotton Does Not Disrupt the Top-Down Forces Regulating the Cotton Aphid in Central China
Source: PLoS One. 2016 Nov 21;11(11):e0166771. doi: 10.1371/journal.pone.0166771 (PMC5117714; doi:10.1371/journal.pone.0166771)

## Conventional cotton

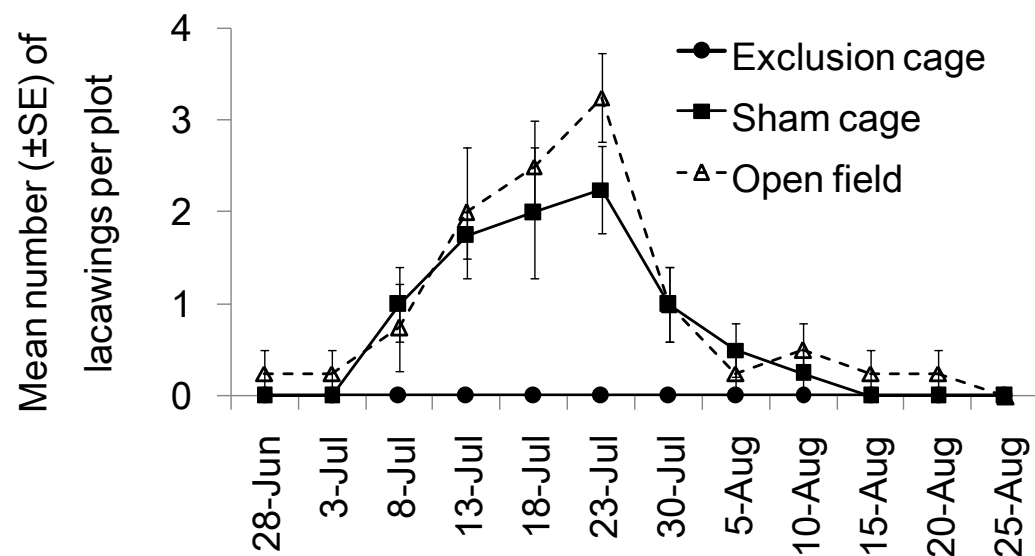

## Bt cotton

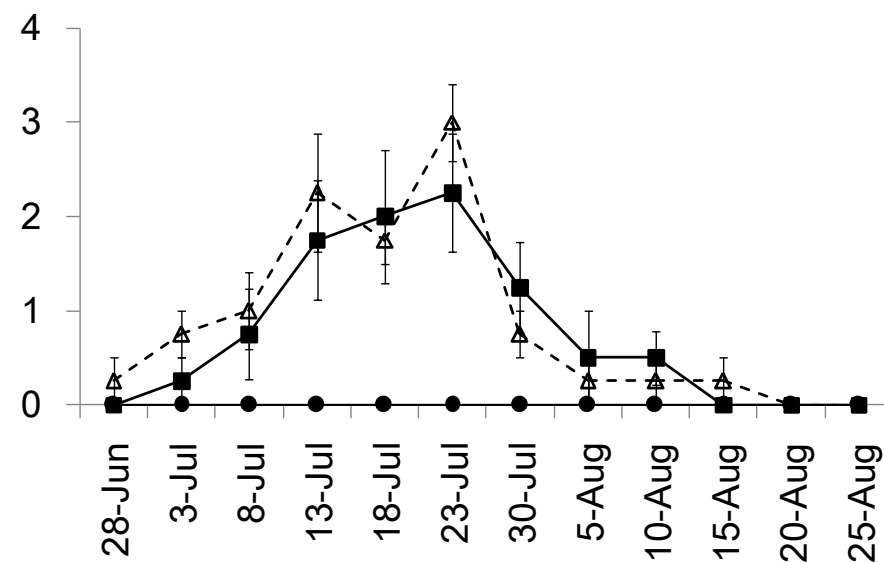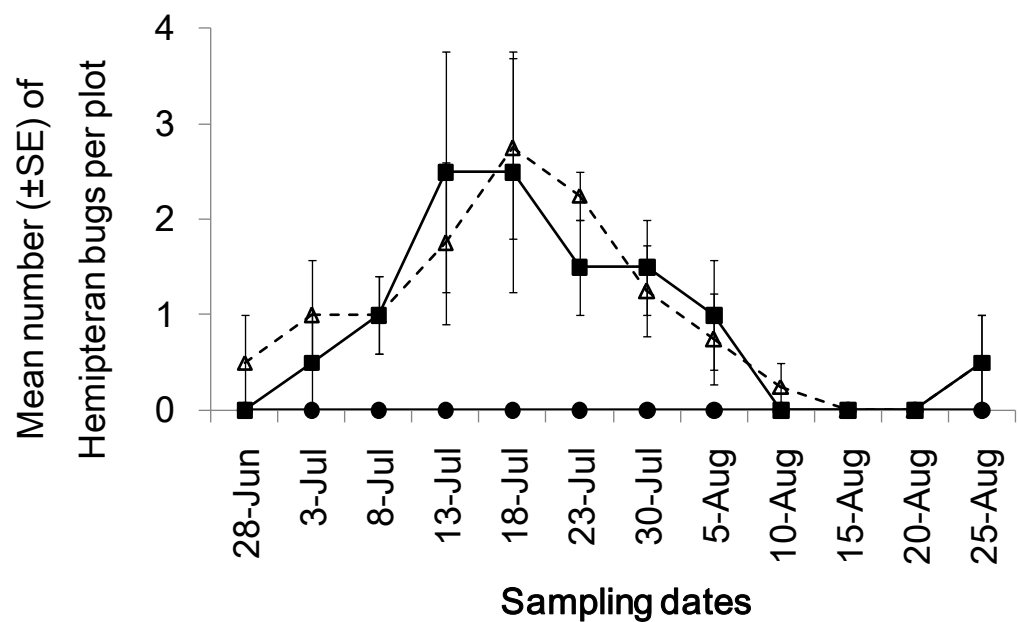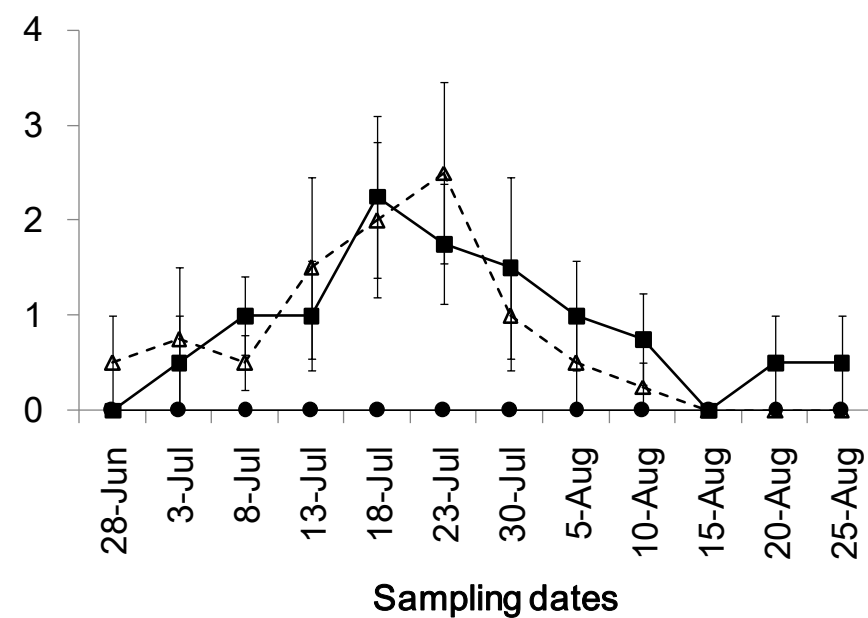

Supplement: S1 Fig — Mean numbers (±SE) of lacewings and Hemipteran bugs per plot in exclusion cages, sham cages and open field plots at HZAU experimental station (Wuhan, China) from late June to late August in 2013. (PDF) [file pone.0166771.s001.pdf]
